# Youth engagement in research: exploring training needs of youth with neurodevelopmental disabilities

**DOI:** 10.1186/s40900-023-00452-3

**Published:** 2023-07-10

**Authors:** Samantha Yimeng Dong, Linda Nguyen, Andrea Cross, Amanda Doherty-Kirby, Jessica Geboers, Dayle McCauley, Alice Kelen Soper, Amanda St. Dennis, Danny Steeves, Natasha Trehan, Jan Willem Gorter

**Affiliations:** 1 Bachelor of Health Sciences Program, McMaster University, Hamilton, ON Canada; 2 CanChild Centre for Childhood Disability Research, McMaster University, Hamilton, ON Canada; 3 School of Rehabilitation Science, McMaster University, Hamilton, ON Canada; 4 Department of Pediatrics, McMaster University, Hamilton, ON Canada; 5 Youth Engagement in Research (YER) Partners/Patient Authors, Hamilton, Canada; 6 Childhood Cerebral Palsy Integrated Neuroscience Discovery Network (CP-NET), McMaster University, Hamilton, ON Canada; 7 Biomedical Science Program, Ottawa University, Ottawa, ON Canada; 8 Department of Rehabilitation, Physical Therapy Science & Sports, University Medical Center Utrecht, Utrecht, The Netherlands

**Keywords:** Youth, Engagement, Patient-oriented research, Disability, Training, Inclusion

## Abstract

**Background:**

Authentic researcher-youth partnerships in patient-oriented research (POR) where the research responds to the needs expressed by youth themselves are essential to make research meaningful. While patient-oriented research (POR) is increasingly practiced, few training programs exist in Canada and none, to our knowledge, are tailored for youth with neurodevelopmental disabilities (NDD). Our primary objective was to explore the training needs of youth (ages 18–25) with NDD to enhance their knowledge, confidence, and skills as research partners. Our secondary objective was to identify the benefits and challenges of engaging youth with NDD in a POR approach.

**Methods:**

Our team of four youth and one parent with lived experience [Youth Engagement in Research (YER) partners] and six researchers engaged in POR to investigate the primary objective via two phases: (1) individual interviews with youth living with NDD and (2) a two-day virtual symposium with focus groups with youth and researchers. Collaborative qualitative content analysis was employed to synthesize the data. Our secondary objective was assessed by asking our YER partners to complete the Public and Patient Engagement Evaluation Tool (PPEET) survey and participate in reflective discussions.

**Results:**

Phase 1 participants (n = 7) identified various barriers and facilitators to their engagement in research and offered suggestions to meet their needs through minimizing barriers and integrating facilitators, which would subsequently enhance their knowledge, confidence, and skills as research partners. Informed by phase 1, phase 2 participants (n = 17) prioritized the following POR training needs: researcher-youth communication, research roles and responsibilities, and finding partnership opportunities. For delivery methods, participants stated the importance of youth representation, using Universal Design for Learning, and co-learning between youth and researchers. Based on the PPEET data and subsequent discussions, YER partners agreed that they were able to express views freely, feel that their views were heard, and that their participation made a meaningful difference. Challenges included scheduling difficulties, ensuring multiple methods for engagement, and working under short timelines.

**Conclusion:**

This study identified important training needs for youth with NDD and for researchers to engage in meaningful POR, which can subsequently inform the co-production of accessible training opportunities with and for youth.

**Supplementary Information:**

The online version contains supplementary material available at 10.1186/s40900-023-00452-3.

## Abstract (plain language summary)

Partnerships between researchers and youth, known as patient-oriented research (POR), are needed to make sure research is meaningful to youth. Our main goal was to explore the training needs of youth (ages 18-25) with neurodevelopmental disabilities (NDD) to enhance their knowledge, confidence, and skills as research partners. To find out, our team of four youth and one parent with lived experience (YER partners) and six researchers completed this project in two parts: 1) interviews with youth and 2) a two-day virtual workshop with youth and researchers. Data from the two parts were reviewed to answer our question. We learned from Part 1 that the needs of participants can be met by providing support and reducing barriers in POR. From Part 2, the top three important topics in partnerships were: researcher-youth communication, research roles and responsibilities, and finding partnership opportunities. Participants in the workshop emphasized having different youth represented, using a framework that allows learning for everyone, and co-learning between youth and researchers in the creation of learning materials. Our second goal was to understand the benefits and challenges of our partnership. To assess, YER partners completed a survey and reflected about their experiences. YER partners agreed on being able to express views, feel that their views were heard, and that their participation made a meaningful difference. Challenges included scheduling difficulties, providing multiple ways to partner, and working under short timelines. Overall, the study described important POR needs for youth and researchers, which can inform future training opportunities.

**Supplementary Information:**

The online version contains supplementary material available at 10.1186/s40900-023-00452-3.

## Background

Patient-oriented research (POR) is defined by the Canadian Institutes of Health Research (CIHR) as a “continuum of research that engages patients as partners, focuses on patient-identified priorities and improves patient outcomes” [1]. Patient stakeholders can be broadly defined as individuals with lived experience with a health condition (including neurodevelopmental disabilities), which includes patients, caregivers, and other family members. For context, neurodevelopmental disabilities (NDD) is generally defined as a heterogeneous group of brain-based conditions, such as Cerebral palsy (CP), Autism Spectrum Disorder (ASD), and Attention Deficit Hyperactivity Disorder (ADHD), that could affect the developmental progress of individuals [2]. As opposed to participation in a research study (i.e., the patient is a subject in a study for data collection), patient partners are actively engaged throughout the research process [1, 3]. Studies have shown multiple benefits of patient-researcher partnerships for both researchers and patients themselves [4].

From the patient perspective, POR democratizes research and ensures that study outcomes are *informed* and *meaningful* [4–6]*.* Meaningful outcomes are those that respond to the needs expressed by patient partners through genuine engagement, which encompass elements such as authentic, non-tokenistic, trust-based, and mutually beneficial relationships between researchers and patients [1, 4, 7, 8]. As such, it is essential that researchers iteratively ask patients about what they consider as important. Research involvement can lead to personal benefits, such as learning new skills, accessing useful knowledge, and feeling empowered through a sense of self-worth by contributing to research that matters to them and their community [5, 6, 9]. In health research, POR can offer value to guide emerging research directions based on patient-identified needs, enhance study recruitment and retention rates, increase the accuracy of results, and broaden the dissemination of research findings to ensure it reaches knowledge users [4, 5, 10]. There is growing recognition of the benefits of POR and increasing efforts to engage with patients and families as partners in research [11–15]. CIHR initiated Canada’s Strategy for Patient-Oriented Research (SPOR) in 2011 to fund and build capacity for meaningful patient engagement [16].

Currently, there are a variety of methods to facilitate engagement. Patients can be involved to varying degrees, for example, at information sessions, attending priority setting events, or taking on greater capacity as a project co-investigator [4, 5]. For stakeholders of disability research, engagement often involves planning and evaluating service delivery interventions (e.g., rehabilitation services) [17]. Identified facilitators for stakeholders, including youth and young adults (hereafter referred to as youth) with NDD and their families, consists of having open communication, clarifying roles, offering training and support, being flexible, and researchers acknowledging youth expertise [9, 17, 18].

Despite efforts to support youth engagement in research, barriers to engagement with youth and researchers continue to persist. Youth have described barriers to engaging in research that include power imbalance between the researcher and patient, navigation of logistics (e.g., medical flares, schedules, geographical distance), and lack of knowledge about the research process and jargon [5, 16]. From the researcher’s perspective, factors that hinder engagement include the time and cost to recruit or sustain authentic engagement, challenges in establishing representative and diverse engaged populations, and difficulties consolidating contrary/varying lived experiences of engaged populations [18, 19]. As one of the core strategies for engagement, SPOR calls for the creation of tools and resources to support research collaboration among patients and researchers, and address the associated barriers that limit effective POR [1, 16].

Previous work has been conducted to respond to the gaps in training for researchers and family partners in NDD and child health research, parent partners and researchers at CanChild Centre for Childhood Disability Research at McMaster University and Kids Brain Health Network co-developed a 10-week online training program: ‘Family Engagement in Research Certificate of Completion Program’ (FER course) [20]. Since 2018, the FER course has been co-instructed by researchers and parent partners. This program was designed specifically for family members (e.g., parents, siblings, grandparents) of youth with NDD and researchers (e.g., trainees, research coordinators, investigators, and clinician scientists) from across Canada and internationally to co-learn the theory and practice of family engagement in child health research.

In August of 2020, researchers and youth patient advisors of an Ontario Brain Institute funded integrated discovery program called the Childhood Cerebral palsy Integrated Neuroscience Discovery Network (CP-NET) held a stakeholder meeting. At this meeting, youth patient advisors discussed the limitations of the FER course, as it was not designed specifically for youth with NDD. They identified the need to develop opportunities that could empower youth with NDD to become more involved in research partnership roles (e.g., co-investigators, collaborators). They also expressed an interest to partner with researchers to develop training materials to equip youth with NDD with knowledge and skills to engage in research as partners. Researchers and youth patient advisors with lived experience of NDD then came together to form a team to address this training gap. Further exemplifying the training need, current POR training resources are broad, and are not designed or responsive to the needs specifically for youth with NDD [21–23]. This study is focused on the training needs of youth with NDD. Findings from this study could inform the development of a future course with and for youth with NDD.

Our research engaged youth with NDD as co-investigators (hereafter referred to as, Youth Engagement in Research (YER) partners for brevity) to address the POR training gaps for this population. The study objectives were:

1. Explore the training needs (content and delivery) for youth (ages 18–25) with NDD to enhance their knowledge, confidence, and skills as research partners.
2. Identify the benefits and challenges of engaging youth with NDD in a patient-oriented research approach.

Although young people, youth, and young adults can be defined anywhere between the range of age 10 to 30, we recognize that the training needs of youth across different ages can look different and we selected the ages 18 to 25 as a starting point for our study [24, 25].

## Methods

### Study design

This study was designed as a qualitative descriptive study to identify and describe the training needs of youth (ages 18 to 25) with NDD, as well as the benefits and challenges of engaging youth with NDD as partners in a research study [26, 27].

To address our primary objective, the following study phases were conducted:

I. Individual semi-structured interviews with youth to explore training needs, barriers, facilitators, and benefits regarding POR.
II. A two-day virtual symposium (September 15th and 25th, 2021) structured as focus groups with researchers and youth with NDD to discuss preliminary findings from Phase I, brainstorm delivery methods and prioritize training topics for the knowledge translation (KT) training materials.

To address our secondary objective, we evaluated our POR approach in our team throughout the study using the Public and Patient Engagement Evaluation Tool (PPEET) [28] completed by our YER partners and reflective group discussions with our team. We also report on our partnership with YER partners using the Guidance for Reporting Involvement of Patients and the Public (GRIPP2) short form [29]. Ethics approval was obtained from the Hamilton Integrated Research Ethics Board (HiREB) on November 16, 2020 to conduct this study.

### Patient-oriented research approach

Our team used a POR approach to design and implement this study. The YER team consists of six researchers and five partners with four youth and one parent with lived experience related to NDD. The YER team was formed from the beginning with the design of this study as our YER partners identified the need to conduct this study to identify the training needs of youth with NDD. Three of our YER youth partners were youth patient advisors with CP-Net who identified the need for this study and expressed an interest to partner in research to identify and address the training needs of youth with NDD. One of our YER youth partners who reached out to the team for opportunities to engage as partners in research. Our team connected with a YER parent partner with an interest in encouraging youth to engage in research. Our YER parent partner is a graduate of the FER course [20] and runs an Instagram account [30] to disseminate information about YER. Two of our YER partners completed courses about engagement in research prior to joining the team. During the final stages of this study and at the time of writing this paper, three of our YER youth partners completed the FER course. Our YER partners collaborated with researchers on a continuum by contributing to the preparation, execution and knowledge translation stages of this research study (see Table 1). Monthly meetings were held on Zoom between August 2020 (inception of the project) to August 2022. Meetings were also recorded, and both the recordings and meeting notes were shared with the full team after the meeting to ensure clear communication. Email correspondence was the primary communication method between meetings to follow-up on action items and gather iterative feedback on documents and decisions. Individual meetings were also held with the student investigator (first author, SYD) to provide opportunities for YER partners to reflect and communicate the level of contribution they wish to engage in for each study phase of preparation, execution, and knowledge translation. These individual meetings took place approximately 3–4 times a year, and additional individual meetings were held as needed. During these individual meetings, the Ontario Brain Institute’s ‘Ways Community Members Can Participate in the Stages of Research’ framework was used as a reference to highlight possible tasks [31]. The Involvement Matrix helped organize the tasks and allow YER partners to identify the level of involvement they hope to contribute. For example, they could have the roles of being a Listener, Co-thinker, Advisor, Partner or Decision-maker (listed in the order of increasing involvement) [32]. SYD administered the Public and Patient Engagement Evaluation Tool (PPEET) with our YER partners, an anonymous 21-item survey containing questions that ask partners to rate agreement to statements or provide comments and feedback [28]. The purpose of completing the PPEET was to give additional opportunities for YER partners to reflect on our group processes and dynamics (e.g., communication and support, opportunity to share views and perspectives, impact and influence of engagement, and final thoughts) [28]. Following the administration of the PPEET and prior to entering the preparation, execution and knowledge translations, our team had reflective group discussions led by first author, SYD, to share our perspectives and experiences of our partnership. Both the PPEET, individual meetings, and group meetings addressed our secondary aim to understand the benefits and challenges of engaging youth with NDD in POR.

**Table 1:** Research activities contributed by YER partners in this project

Research Stage	Examples of activities with YER partners
Preparation	Developed the idea and concept for the study Informed the study protocol (research questions and methods) Shaped interview questions so that they were framed appropriately for youth with NDD (e.g., plain language) Planned the virtual symposium, such as the breakout room questions and activities Recorded themselves for the recruitment videos Provided feedback on ethics and grant application that was incorporated into final drafts
Execution	Recruited participants by sharing the recruitment poster and videos on social media and word-of-mouth Conducted interviews Co-hosted and facilitated the two-day virtual symposium Conducted qualitative data analysis collaboratively by coding and engaging in group discussions
Knowledge Translation	Provided feedback on abstracts, manuscript, and posters Co-developed the prototype of training materials (e.g., video and infographic) Identified opportunities and co-presented findings at research conferences (CP-NET Science and Family Day, 2021, Children’s Healthcare Canada Annual Conference, 2021)

We provided YER partners honorariums, gifts, and bonding experiences to express our appreciation for their time and hard work. We had a discussion with each YER partner and asked how they would like to be compensated. We provided options for compensation, including an honorarium based on guidelines from a pan-Canadian childhood disability research network [33], as well as customized sweaters, and gift cards for restaurants where YER partners can order food and join for a virtual get-together on Zoom. We offered different options for the customized sweaters, and YER partners selected the design that they preferred. These different options for compensation were a way to show appreciation for the engagement of each YER partner, which aligns with experiences that other research teams have had when providing compensation for youth and family partners in research [34]. Our bonding experiences extend beyond the purpose of compensation, and was a way for us to build connections, trust, and a sense of belonging with the team.

## Data Collection

### Phase I: Individual interviews

The sample size for this phase was 10 participants. This sample size was informed by the literature and our partners to be adequate and feasible for describing POR training needs of youth with NDD [35]. These interviews were led by the student investigator and first author, SYD, through Zoom [36]. We also invited our YER partners to be involved with the interviews with SYD. Each session took one hour and included nine questions (see Additional File 1). With consent from participants, the sessions were recorded and auto-transcribed via Zoom [36]. SYD manually checked the accuracy and de-identified the transcripts before data analysis.

### Phase II: Virtual symposium with focus groups

A two-day virtual symposium was held on September 15^th^ and 25^th^, 2021. The symposium lasted for 2 h for each day for a total of 4 h. Details of the schedule for each day is presented in Additional File 2. The purpose was to discuss preliminary findings from the interviews from Phase 1 and conduct a series of focus groups to brainstorm delivery methods and prioritize training topics for the KT or training opportunities. Co-authors SYD and ASD co-hosted the symposium. Multiple team members, including our YER partners, facilitated the focus groups that were conducted in breakout rooms on Zoom [35–37]. Google Jamboard, an anonymous and accessible virtual whiteboard, was used alongside live discussions to record any thoughts and ideas from participants [38]. The symposium with the focus groups were recorded and auto-transcribed via Zoom before data analysis [35–37].

## Participants

### Phase I: Individual interviews

Youth with NDD were recruited to participate in semi-structured individual interviews through various networks (e.g., youth advisory councils from Children’s Hospitals and Children’s Treatment Centres across Canada, Easter Seals, CHILD-BRIGHT Network, Kids Brain Health Network, CanChild). Recruitment posters and videos were circulated among these networks via email, newsletters, and social media. Recruitment videos were co-created by our team with YER partners identified using this strategy to increase accessibility and reach a wider audience. In addition, the ‘snow-ball sampling’ method was implemented in which participants were asked to recommend other youth to participate in this study [39]. Eligible youth participants were English-speaking (including via alternative communication devices and methods) between 18 and 25 years old. Upon expression of their interest to participate in the study, SYD sent an informed consent form and demographic questionnaire via email prior to scheduling the individual interviews held on Zoom [36].

### Phase II: Virtual symposium with focus groups

Researchers and youth with NDD were recruited to participate in a two-day virtual symposium with focus groups through targeted email correspondence, as well as the ‘snow-ball sampling’ method [39]. The same eligibility criteria for the individual interviews were applied to youth participants for the virtual symposium. Interested participants were sent an informed consent form and demographic questionnaire via email before providing the Zoom links for both symposium dates [36]. Participants from Phase I were invited to participate in Phase II with the virtual symposium with focus groups. Participants had the option of attending day one, day two, or both. An electronic package was developed and sent to participants before the symposium to clarify the purpose and itinerary. The package also provided key definitions and acronyms relevant to POR and NDD.

### Data analysis

Conventional content analysis was conducted for the data collected from the interviews and the virtual symposium [40]. This type of content analysis was used to identify the codes and categories from the data without using pre-conceived categories [40]. We invited all members of our YER team to be involved with data analysis, and there were specific team members who expressed an interest in analyzing the data in detail. We then formed a subgroup with researchers (SYD and LN) and YER partners (ADK and JG) to code the data. A researcher on our team (LN) with experience in qualitative data analysis provided training by creating a short video tutorial to describe the process of coding. This video tutorial included examples of how to code a transcript based on the literature [40–42]. Each transcript was independently coded twice, once by SYD and once by another member of the subgroup [42]. The full de-anonymized transcripts were imported and the coding process was conducted using Dedoose, a mixed-method data analysis software [43]. The initial codes were individually developed based on reading and re-reading the transcripts [41, 42]. The subgroup then held discussions to refine the codes and combine codes that were similar to each other. The codes were categorized into barriers (i.e., difficulties to engage in research) and facilitators (i.e., supports to engage in research) related to individual and contextual factors, and all code definitions were listed in a preliminary codebook [42]. Individual factors are factors that are more directly related and/or tangible to address for youth, whereas contextual factors are related to the status quo environment or societal conditions. The categorization of these factors were based on the theoretical lens from Bronfenbrenner’s ecological theory, where individual factors exist within the broader context of the macrosystem with the environment and society [44]. The preliminary codebook was circulated to the full team to refine codes and definitions [42]. SYD then revised and finalized the codebook [42]. After developing the codebook, two reviewers coded each transcript and had discussions to reach agreement during the coding process [42]. When needed, a third YER partner (ASD) had the role of a reviewer to provide their perspectives to resolve any discrepancies between coders [42].

### Trustworthiness

Several strategies were employed to enhance trustworthiness of the data [45, 46]. As a common qualitative method, respondent validation was conducted during the interviews in Phase I where the interviewer(s) summarized the information that participants shared at the end of the interviews [47]. Participants could then have the opportunity to clarify and/or add additional information to the interviews [47]. Member checking was also conducted after the virtual symposium to ensure proper representation of participant’s opinions and perspectives, whereby a summary sheet of the findings was emailed to participants for feedback (see Additional File 2) [48]. We received positive feedback and confirmation of our findings from participants. Another strategy that was used throughout the study was reflexivity, in which all team members including YER partners were encouraged to create reflexive notes to share their experiences, opinions, and thoughts [49, 50]. For example, our subgroup of researchers and YER partners involved with coding the data created reflexive notes to document their thoughts while reviewing and analyzing the data [49, 50]. An audit trail was kept throughout the study to document our meeting discussions and decisions that were made as the study was designed and conducted [46].

## Results

### Demographics

Phase I: Nine youth expressed interest in participating in the study and seven met the inclusion criteria. There was representation from youth with ASD, ADHD, and CP; as well as a distribution of individuals who identified as men, women, and another gender (see Table 2).

**Table 2:** Focus Group and Interview Demographics

	%
Youth (ages 18–25)	100
ASD	14
CP	29
ADHD	43
ASD & ADHD	14
Gender	
Men	29
Women	42
Another gender	29

Phase II: In addition to the YER team, a total of 17 youth with NDD and researchers participated in the two-day virtual symposium. Of the participants, 10 were youth with NDD (ASD, ADHD, CP) and seven were researchers (i.e., coordinator or faculty member) or trainees (i.e., a graduate student or postdoctoral fellow) in the field of POR and/or NDD (see Table 3).

**Table 3:** Virtual symposium demographics

	n	%
Total Participant	17	100
Youth (age 18–25)	10	59
ASD	1	6
CP	4	24
ADHD	3	18
ASD and ADHD	1	6
CP and ASD	1	6
Researchers/Trainees	7	41
Researcher	2	12
Trainee	4	24
Other	1	6
Gender		
Men	5	29
Women	11	65
Other gender	1	6

### Training needs for engagement in research

**Fig. 1:**
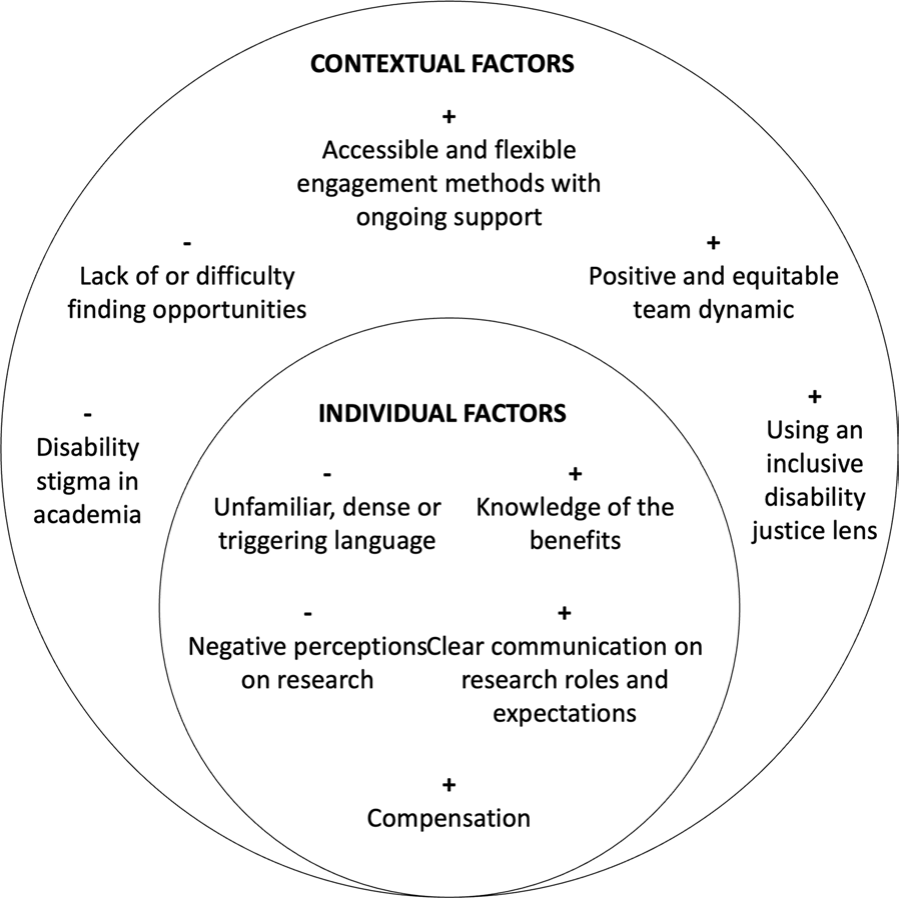
Individual and contextual barriers (−) and facilitators ( +) in POR based on direct content analysis

Qualitative data from Phases I and II identified the need for training opportunities to address: i) researcher-youth communication (including language barriers); ii) clear research roles and responsibilities; and iii) knowledge about where to find partnership opportunities. Within individual and contextual factors, barriers and facilitators exist, which are indicated by the minus and plus signs respectively in Fig. 1.

Individual factors were factors that individuals can directly change in the short-term during a research project. For example, a participant described how the lack of information about accommodations prevented them from being involved in research positions:

“It kind of just comes back to like lack of accommodation. Like I know how I exist in spaces. I know what accommodations I need to have to be at a level where I can deal with heavier workloads… [but] you know approaching a new opportunity and not knowing what kind of accommodations are possible can and has prevented me from applying for positions entirely.” (Participant 7).

It is important to understand the perspectives, motivations, and interests of youth with NDD, which researchers can do by having discussions regarding research roles and expectations, benefits about being engaged in research, and compensation. If youth with NDD are interested in developing specific skills to acquire knowledge and develop skills in POR, researchers can also seek opportunities to offer training in these areas to the whole team.

Contextual factors were factors within a broader environment, such as society and institutions, that indirectly affect the research project and may not be changed immediately during a project. For example, participants shared about the lack of or difficulty finding opportunities. A participant describes this difficulty:

“You often have to follow like either your local autism association or organization to be aware or to know of studies. So they're not you know, like you, kind of have to look for them to find them. So I think there's also that they're not made accessible to everyone.” (Participant 6).

Participants described support that they need to be involved in research, such as having a welcoming, positive, and equitable team environment. One participant identified how a safe space could look like:

“… where it's saying hey this is a particular safe space. These are the things that we're doing beforehand to create the safe space. If you have any questions or like this crosses a line or something like that or you feel uncomfortable. Here's how you exit in the easiest way possible.” (Participant 3).

To address the individual and contextual factors to promote engagement with youth with NDD in research, questions for the research team to consider can include:

- What are the research roles and expectations?
- Will engagement be accessible and flexible (language, multiple methods of communication, timeline, etc.)?
- Will there be ongoing support, communication and opportunity to self-advocate?
- What are the benefits of engagement in research?
- Will there be compensation or recognition of contributions?

When asked how youth can feel more confident engaging in research as partners, many reported that being equipped with the knowledge and skills can empower them.

Participants also proposed that confidence can be enhanced if the research landscape shifted toward an inclusive disability justice model and avoid framing disabilities as a deficit. One youth recommended Sins Invalid as a resource to learn about disability justice principles [51]. By seeing disabilities through this lens, researchers and youth can work together to promote inclusion in society, as opposed to ‘fixing’ people with disabilities [51]. One participant strongly conveyed how deficit-based approaches in disability research makes engaging difficult:

“That's just been a very traumatizing and difficult experience, where [researchers] have not felt like taking an interest in autistic people in order to help and shape the system and everything as it is to fit us, but to just fix us.” (Participant 6)

To further illustrate the need for a paradigm shift, some participants expressed feeling that they must mask their disability (when possible) to avoid being stigmatized in academic and research settings. If academia can foster an inclusive disability-positive and strengths-based mindset, this does not have to be the status quo:

"You kind of have to mask it when you're approaching research because in like research context and academic context as soon as you start saying hey you know I'm disabled, your credibility kind of goes away." (Participant 3)

The perceived need from youth with NDD to mask their disability can be a barrier to engage in research. There is a need to foster opportunities and spaces where youth with NDD are valued for their lived experience expertise and can bring their true authentic selves when engaging in research.

In Phase II, the virtual symposium discussions between youth and researchers further prioritized training needs and highlighted possible formats for training opportunities. The following training topics were prioritized: (1) communication training between youth and researchers, (2) research roles and responsibilities, and (3) finding research partnership opportunities. Specific content and delivery methods proposed for each topic can be found in Additional File 2. In terms of training delivery methods, Universal Design for Learning (UDL) was suggested as a potential framework [52]. In the spirit of UDL, participants suggested multiple methods of delivery: videos, infographics, and interactive modules among other formats. Many participants came to a consensus that having a variety of platforms ensure accessibility:

“Maybe have different options because different people find different things accessible. Like some people are more auditory-visual learners so like training videos maybe. But other people may learn better from reading. So I think it depends on the individual and it's best to have a variety.” (Participant 6)

Furthermore, representation of youth in the creation/delivery of training material was deemed important and necessary. Participants believe this may benefit engagement due to relatability. Youth presentation can also mitigate any potential patronizing tone (e.g., if a non-NDD researcher is “telling” youth with NDD what to do). However, participants also questioned the notion of training. They stated that youth should not feel obligated to have research skills before POR engagement because an area of expertise they bring is their lived experience and that research skills can be learned along the way. In addition, the idea of POR training *only* for youth may not be fair, as it implies that the onus to enhance POR is on them. Instead, participants agreed that researchers should also take initiative and co-learn how to conduct effective POR (Table 4).

**Table 4:** Prioritized training needs and potential training formats

Prioritized Training Needs (Topics)	Potential Training Formats
Communication training between youth & researchers Research roles & responsibilities Finding research partnership opportunities	Video(s) with a person speaking to you Whiteboard animations with voiceover Infographic, Documents, Checklists Mentorship, Personal Check-Ins Online Interactive Modules Self check-ins, activities, and reflections Simulations (e.g., scenarios + solutions)

Lastly, two key messages permeated both Phase I and II, which are communication and self-advocacy. To address communication barriers, youth participants emphasized the importance of using plain language when non-NDD researchers partner with youth with NDD in research. Unfamiliar language (academic jargon and acronyms) and dense academic writing styles can make research intimidating, causing youth to hesitate to engage in research. Instead, researchers should aim to define jargon and acronyms, and use plain language whenever possible. Other language considerations include asking youth about their preference for referral (e.g., person-first or identity-first language) and which terminologies should be avoided. For example, one participant knew other autistic individuals who found the word “intervention” triggering, which can have a negative connotation associated with having deficits that need to be “fixed”. One participant highlighted how accessible language can address the power dynamics in academia:

"I think language in [and] of itself is huge because language is tied to so many things like class and disability… So I think like just having more accessible language that the average person can read and understand.” (Participant 7)

With regard to self-advocacy, participants from both phases want youth to know that they *can* and *should* advocate for their accessibility needs and accommodation in research partnerships, especially if researchers are not initiating these conversations. One participant wished that someone would have told them that they can speak up in research partnerships:

"I think it would still be really good for someone to even tell me like okay you are allowed to speak up for yourself and you're allowed to tell me when you're feeling overwhelmed and these are your rights and this is what you can do when these situations are happening. Because that was never laid out for me before." (Participant 5)

Overall, youth with NDD identified barriers to minimize and provide facilitators to support them in partnerships with researchers. Perspectives from both youth and researchers provided direction for POR training topics and delivery methods to co-learn together.

### Patient-Oriented research outcomes

The role of YER partners (n = 5) evolved over time as the team entered different stages of research. YER partners expressed their thoughts on the group processes and dynamics via the PPEET survey (see Additional File 3). Based on the results of the survey, all partners believed that they had a clear understanding of the study purpose, that the supports they needed to partner in research were available, and that they had enough information to carry out their role as a partner. Our partners generally believed the strength of this project lies within the clear communication and organization, the positive and safe culture fostered, and hearing diverse perspectives from both researchers and youth. Personal benefits to YER partners were being able to express views freely, feeling that their views were heard, and that their work makes a meaningful difference to them and the research. Our YER partners provided open-ended responses to the survey about their experiences partnering in this study. For example, a partner described the benefit of being better informed about youth engagement in research as a result of our partnership: “I feel like I am learning so much. I consider many on the team to be mentors.” Another partner shared how they were able to make a difference: “I have helped to make sure that our project and the materials we produce are accessible to all stakeholders. I also bring a lived experience with disability.” Partners agree that engagement was a good use of their time and that they felt satisfied with their contribution.

While there were benefits to engagement based on the survey results, YER partners also identified challenges in engagement. These challenges included scheduling difficulties, ensuring multiple methods for engagement, and navigating tasks in a short period of time. We proactively addressed these challenges throughout our partnership by communicating flexible deadlines, and offering individual meetings when team meetings were missed due to extenuating circumstances (e.g., medical appointments) or if partners would like more opportunities to share ideas. In addition, Google Jamboard, an anonymous and accessible whiteboard, was used during meetings as another method to gather perspectives [38]. YER partners mentioned this being a helpful addition to meetings, as sometimes they wanted to bring up new topics but did not want to interrupt the conversation taking place. The use of the Jamboard was also useful to gather additional ideas or feedback after meetings. Overall, we had a positive experience and iteratively welcomed feedback from team members to improve our POR approach.

## Discussion

This study aimed to determine the training needs of youth with NDD to enhance their knowledge, confidence, and skills as research partners, as well as to identify the benefits and challenges of engaging in a POR approach. To investigate these objectives, our research team collected qualitative data from interviews in Phase I and a two-day virtual symposium with youth and researchers in Phase II. From both phases, we identified training needs and potential training delivery methods. By engaging people with lived experience as co-investigators on this project, we have our own insight into the benefits and challenges of POR.

### Training needs

Our study identified several POR training needs, based on engagement barriers and facilitators, which include understanding research roles and expectations, accessibility and flexibility, ongoing support, benefits, compensation, and where opportunities exist. Youth and researchers during the virtual symposium prioritized the following: researcher-youth communication, research roles and responsibilities, and finding partnership opportunities. We share how these priorities are similar to and expand on findings from existing literature, and offer strategies on how to address these priorities based on the experiences of our partnership in this study. By acquiring the knowledge and skills associated with training needs, youth with NDD can feel more equipped, empowered, and confident in collaborating with researchers as partners. As such, the findings of our study can serve as content considerations for future training opportunities.

## Researcher-youth communication

We recognize that clear, accessible, and ongoing communication is at the core of POR partnerships. According to a recent scoping review on studies where researchers engaged youth with disabilities, including NDD, and their families, it is widely encouraged that early and ongoing reciprocal communication occurs [18]. Another study, where one member of the team was a youth with CP, also emphasized the importance of communication between researchers and youth to clarify roles and set mutual expectations before engaging in research [9]. Similar to what participants in our study have expressed, Cavens et al. recommend that researchers continually provide various modes of communication to sustain long-term interest, such as offering additional remote one-on-one opportunities to freely share ideas [9]. While the study conducted by Cavens et al. focused on youth ages 8–18 years old with cerebral palsy [9], our findings were similar in that researchers that create the space for consistent and open communication can enable youth to self-advocate concerns related to accessibility and accommodations. The other piece to communication is being mindful of accessible and inclusive language. Language barriers in engagement are commonly noted in the literature, which include unfamiliar, dense, and academic jargon [5, 53, 54]. Camden et al.’s scoping review of studies that involved engaging people with disabilities and their families stated that “scientific language and research materials needed to be adapted to avoid jargon, ensuring everyone understood and felt comfortable and confident to engage in meaningful dialogue” [17]. It is important to consider how to communicate in ways that would provide opportunities for youth with NDD to actively be involved in research as a partner alongside researchers. We suggest strategies to promote clear communication between youth and researchers: ensure accessibility (e.g., documents and audio recordings of documents, offering video recordings of meetings, using plain language and avoid the use of jargon); do not be afraid to ask for clarification (e.g., youth can ask for accommodations, and researchers can ask how these accommodations can be provided); and respect the experiences and expertise that all team members bring. In brief, strategies that incorporate ongoing communication through multiple modes of communication, utilize lay language, and valuing all expertise in ascertaining POR engagement are recommended. We encourage researchers to consider how the application of these strategies could allow for youth with NDD to become partners and lead research similar how we modeled our partnership of engaging with our YER partners in this study.

## Research roles and responsibilities

We first acknowledge that youth do not need to be fully equipped with specialized research knowledge and skills, as one area of expertise they bring to the research team is their lived experience. In the current landscape, partners are often encouraged to engage in roles related to interpretation and KT, as it is deemed a feasible way to engage and ensures that KT will be tailored and useful for knowledge users [55, 56]. With that said, partners should have autonomy and room to discover new roles because predetermined roles and expectations can be perceived as a barrier [57]. As such, it is important to discuss, break down tasks, identify levels of interest, and provide support wherever necessary. YER partners reported that periodic use of tools, such as the Involvement Matrix, can help define roles and the extent of responsibilities throughout the research process [32]. In addition, using guides, such as the Ontario Brain Institute’s ‘Ways Community Members Can Participate in the Stages of Research’ or the ‘Knowledge-to-Action’ framework, can aid in identifying possible roles at different stages of the research process [31, 55]. As mentioned in the literature, partners may not have familiarity with specialized research skills, such as data collection and analysis; however, these skills can be learned if resources and training are provided [17, 54, 58]. Strategies mentioned during the symposium include creating resources in multiple formats (videos, infographics, modules), skill-building workshops, and mentorship (researcher-youth or youth-youth). An example of training skills during the research process can be found in our team, where LN created a video outlining qualitative study design and the coding process for YER partners that were interested in assisting with data analysis (video link is available upon request). Thus, researchers should continuously support partners to explore roles that they would like to have throughout the stages of research, in addition to the valuable lived experience expertise they bring.

## Finding partnership opportunities

Participants identified how inaccessibility or the lack of awareness of partnership opportunities often hinder or prevent POR engagement. Low awareness has also been reported in studies about POR regarding youth with disabilities and their families [17, 59]. Virtual symposium participants suggested finding creative strategies to promote visibility and centralize opportunities. One idea is to create a national initiative that centralize postings (similar to the ReachBC database), but specific to POR partnerships for youth with NDD [60]. Other ways to cultivate relationships between researchers and youth could be through social media [18], perhaps by profiling both researchers and youth interested in POR engagement. It often only requires one opportunity to open the doors, as partnerships can evolve into long-term collaboration and lead to networking, where new projects may emerge. Three of our YER partners are a prime example of such long-term engagement, where their involvement in CP-NET led to this project. To create inclusive spaces for youth to engage as partners in research, it is important to consider the application of models, such as the social and biopsychosocial models, that move away from the medical model of disability. Historically, the medical model of disability focused on “fixing” the impairment of an individual [61–63]. This perception of “fixing” the disability has led to experiences of stigma [63], and some participants in this study have described the perceived need to mask their disability. We recommend and also apply both the social and biopsychosocial models in our partnership, with the biopsychosocial model and social model. The biopsychosocial model with the International Classification of Functioning, Disability and Health (ICF) framework [64], which has been translated into lay language with the F-words [65], can help to consider the different factors that are part of an individual’s life. Youth are growing up in multiple environments, and the research and team environment can have a bidirectional process with youth where they influence each other [66]. Our team also applied the social model of disability, which describes how the disability is viewed as part of the identity of an individual and it is the environment that causes the disability [61–63]. In our partnership, we developed rapport to build a sense of team cohesion and a welcoming space for our YER partners to ask for accommodations. In all, creating better strategies to raise awareness and direct youth to opportunities in inclusive research spaces can promote POR partnerships.

### Training material delivery method

Key considerations when developing training opportunities include the representation of youth with NDD in training materials, creating opportunities for youth and researchers to co-learn, and consulting the UDL framework. Representation matters as having people with lived experience leading conversations can reduce the ‘cultural’ barrier between researchers and youth [17]. By having youth with NDD co-design and/or deliver the training with researchers, we can bridge the divide and avoid patronizing undertones (researchers “telling” youth what to do), as pointed out by study participants. Several modes of delivery were suggested by participants such as videos, infographics, checklists, mentorship, and online modules. Providing multiple formats is consistent with UDL, an evidence-based framework developed by researchers at the Center for Special Applied Technology [67]. UDL aims to efficiently address a variety of learning needs, regardless of ability, by designing a curriculum centered on having multiple means of engagement, representation, and action/expression. A study demonstrated that the UDL framework aligned well with the needs of students with disabilities [68], rendering it as a useful guide for designing accessible and flexible training materials.

### POR benefits and challenges

Engaging in a POR research team throughout the research process has been beneficial for all members of our research team, which aligns with the benefits found in the literature. Partners provided insider knowledge from their lived experience, which enriches the entirety of the research process [59]. In the context of data analysis and dissemination, partners aid in the interpretation of results, provide credibility, and improve the accessibility of information [18]. As was expressed by YER partners, engagement can be meaningful and empowering as it provides an opportunity to influence the direction of research that impacts them and their community [9, 17, 59]. It is also acknowledged that services co-developed with youth partners are more acceptable and responsive to their needs [54].

A challenge of POR was the additional time and financial resources to sustain engagement, which other studies have also reported [59]. For our team, difficulties that arose included not being able to attend all meetings due to scheduling difficulties and ensure adequate methods for youth engagement. We also experienced the challenge of when our YER partners raised many ideas for this study, and we had to ensure that this study was feasible to complete based on the availability of our budget and time. As outlined by other studies, these issues can be circumvented by providing multiple ways to engage, including providing lay summaries or videos via email correspondence, and additional one-on-one meetings and team discussions to discuss the study plans [9, 17]. In our study, we also had discussions about future directions of our work and the interest of our YER team to continue our partnership in research. At the time of writing this paper, we are continuing to engage with our YER team to consider the next steps of our work. Ongoing discussions are important to ensure that there is clear communication about the study plan and future directions. Overall, we recommend incorporating POR in more disability research practices, as in our experience the benefits outweigh the challenges.

### Recommendations

The following recommendations are for researchers interested in POR, as informed by our qualitative findings, experience engaging in POR as a team, and the literature:

1. Ensure early, ongoing, and accessible researcher-youth communication
2. Support and provide opportunities for youth to discover research roles and learn skills
3. Dedicate time and resources to build team rapport and sustain engagement
4. Create strategies to promote awareness and centralize opportunities for youth with NDD
5. Consider multiple methods when designing and delivering training materials, and throughout the process of engaging with youth in research
6. Share personal experiences and reflections after engaging in POR practices

### Strengths and limitations

The strength of this study lies in the collaboration with YER partners at every stage of the research process, which emphasizes inclusion as the hallmark of POR. A contributor to our team’s cohesion was the length of time we had to build rapport. We held monthly team meetings and individual check ins, in which time was taken to connect with one another, beyond workplace formalities. Such was initially prompted through meaningful icebreaker questions, for instance “What is one rose (highlight) and one thorn (challenge) from this week?”. With time and familiarity, our connection and cohesion strengthened organically, which provided a safe space for our team members to honestly exchange thoughts, iteratively reflect and thereby making decisions together. Our YER partners felt safe to ask for accommodations that could benefit all team members (e.g., audio recordings of documents). This safe space also allowed for clear communication where our YER partners had clear expectations about their roles and could choose what activities they wanted to contribute to the study. During the preparation of this study, partners helped co-design the research protocol to ensure that the goals are relevant and meaningful based on their NDD lived experience. As we entered the data analysis phase, we first intentionally discussed the analysis strategy as a team, then inquired about individual roles. Two YER partners co-analyzed and interpreted the qualitative data and a third YER partner provided their perspectives to resolve any discrepancies between coders. The perspectives from our YER partners during data analysis were valuable in recognizing varying interpretations, which prompted rich discussions before reaching a shared decision. Our team also demonstrated shared decision-making. For example, our YER partners chose which conferences they would like to present at, the content that they would like to include in the abstract submission, and the presentation format. The varying roles that our YER partners had varied fluidly through each phase, based on their choice and interest. The research culture that we adopted was not ‘strictly business’, but rather we connected with each other as people.

A limitation of this study is the small sample size and lack of diversity in the population. Although this study explored the unique perspectives of a specific group of youth with NDD, a wider and more diverse sample could help to apply the findings and develop training materials that would be applicable to a broad range of disabilities outside of NDD. For example, this study did not capture the experiences of youth with intellectual developmental disabilities. In addition, information about racial and ethnic backgrounds was not collected from participants. All of these factors could intersect with NDD and influence an individual’s experience in research partnerships. Thus, the inclusion of a more diverse and representative sample would strengthen future studies in this field.

## Conclusion

This study identified crucial training needs for youth with NDD and researchers to engage in meaningful POR. The qualitative findings highlighted the importance of setting the stage for a positive and inclusive research culture by addressing training needs centered on research-youth communication, research roles and responsibilities, and finding engagement opportunities. Through our partnership with individuals with lived experience, we recognize the value of POR and provide recommendations for navigating POR in practice. We encourage more researchers to provide opportunities to involve youth in research partnerships and to share their experiences and lessons learned. Our findings can be used to inform the development of co-training opportunities for researchers and youth with NDD. With that, a formal evaluation of POR strategies and future training opportunities will be needed. We ultimately aim to expand the practice of effective POR to ensure that research truly embodies the disability rights mantra “nothing about us without us”.

## Supplementary Information

**Additional file 1**. Focus group questions.

**Additional file 2**. Virtual symposium summary.

**Additional file 3**. Partner responses to the public and patient engagement evaluation tool (PPEET).

## Data Availability

The authors confirm that the data supporting the findings of this study are available within the article and its supplementary materials.

## Abbreviations

ADHD: Attention deficit hyperactivity disorder
ASD: Autism spectrum disorder
CIHR: Canadian Institutes of Health Research
CP: Cerebral palsy
FER: Family Engagement in Research
GRIPP: Guidance for Reporting Involvement of Patients and the Public
ICF: International Classification of Functioning, Disability and Health
KT: Knowledge translation
NDD: Neurodevelopmental disabilities
POR: Patient-oriented research
PPEET: Public and Patient Engagement Evaluation Tool
SPOR: Canada’s Strategy for Patient-Oriented Research
UDL: Universal Design for Learning
YER: Youth engagement in research
