# Supplementary material for: Youth engagement in research: exploring training needs of youth with neurodevelopmental disabilities
Source: Res Involv Engagem. 2023 Jul 10;9:50. doi: 10.1186/s40900-023-00452-3 (PMC10332095; doi:10.1186/s40900-023-00452-3)
Supplement: Supplementary file 1 — Additional file 1. Focus group questions. [file 40900_2023_452_MOESM1_ESM.docx]

**Youth Engagement in Research: Exploring Training Needs**

**FOCUS GROUP QUESTIONS**

**Introductory Statement** (To be said prior to the commencement of the focus group):

We’d like to thank you for participating in this focus group. Your time and feedback are greatly appreciated. Before we begin, we’d like to provide a brief overview of the purpose of this focus group and this research project.

The purpose of this focus group is to gain deeper insight from youth and young adults with neurodevelopmental disabilities about what type of training could support their engagement in research. When we refer to (patient) engagement in research, we are talking about *partnering* with researchers throughout various stages of the research process (screen share OBI framework). Partnering in research is different from being involved in research as a *participant*. As a research partner, you may be engaged in research as a patient advisor or a co-investigator, whereby you are involved as an equal member of the research team. Currently, on our research team we have 4 youth research partners that has shaped this project from the start.

Through these focus groups/conversations, we hope to gain a better understanding about the challenges to engage in research and what you believe is necessary to support your engagement in research. These insights can inform the development of training materials that would address those identified challenges and training needs. With training materials on youth engagement in research, we hope to support youth with neurodevelopmental disabilities to gain the knowledge, skills, and confidence necessary to engage as research partners.

We will be taking notes as we speak, but in order to ensure accuracy is it okay with you if we also record the Zoom meeting so we don’t miss anything?

*(Please note: This is a flexible guide. In addition to the questions below, participants may also be asked additional questions that emerge during the conversation, based on how they have answered a previous question.)*

1. Before we begin, do you have any questions about the purpose of this focus group/conversation, or the purpose of the Youth Engagement project as a whole?

2. How would you define being a research partner (and the different roles in research)?

*a.* *Probe: What do you think are the differences between a research partner versus a research participant?*

*b.* *Probe: Do you have any experience as a research partner? If so, please elaborate on your past experience(s).*

c. *Probe: What do you think are the benefits/values of being a research partner?*

3. Are there things that make it hard for youth to engage as research partners based on your own experiences or those of others (youth with neurodevelopmental disabilities)?

*a.* *Probe: What might make youth feel unsure to engage in research as a partner?*

b. *Probe: How might these factors affect how you engage in research?*

4. What do you think is necessary for youth to know before engaging as research partners?

*a.* *Probe: What types of knowledge would help youth as research partners (e.g., knowledge about the research process/skills, advice from experienced research partners, etc.)?*

*b.* *Probe: What questions might youth have about research engagement?*

c. *Probe: What might make youth curious or interested to partner in research?*

5. What type of training material(s) would best deliver the knowledge needs you mentioned?

*a.* *Probe: Infographics, Videos, Presentations, Podcasts, Interactive Modules, Experiential Learning (problem-based learning), etc.*

b. *Probe: How can we make the material more accessible for you (e.g., alternative formats, increased font size, preferred font, image descriptions, captions, preferred font, etc.)?*

6. What skills would you want to develop to support your engagement in research?

*a.* *Probe: Are there any skills you would need for the preparatory/beginning stage of research (e.g., agenda setting/setting relevant research topics, designing a study)*

*b.* *Probe: Are there any skills you would need for the execution of research (e.g., recruitment, data collection, data analysis/interpretation)*

*c.* *Probe: Are there any skills you would need for the knowledge translation stage of research (e.g., sharing results through posters, graphics, videos, etc.)*

7. What type of training material(s) would best help with the skills you need?

*a.* *Probe: Infographics, Videos, Presentations, Podcasts, Interactive Modules, Experiential Learning (problem-based learning), etc.*

b. *Probe: How can we make the material more accessible for you (e.g., alternative formats, increased font size, preferred font, image descriptions, captions, preferred font, etc.)?*

c. *Probe: How much experience would you need for each skill (e.g., having knowledge/context about the skill, performing the skills, etc.)*

8. What would help you to become more confident in engaging as a research partner?

*a.* *Probe: Would hearing the perspectives of other youth research partners be beneficial for youth?*

*b.* *Probe: Would hearing the perspectives of researchers be beneficial for youth?*

*c.* *Probe: What could researchers do to help (e.g., ideal work environment to foster, what should be communicated, how should they accommodate, etc.)?*

9. What type of training material(s) would make you feel more confident as a research partner?

*a.* *Probe: Infographics, Videos, Presentations, Podcasts, Interactive Modules, Experiential Learning (problem-based learning), etc.*

b. *Probe: How can we make the material more accessible for you (e.g., alternative formats, increased font size, preferred font, image descriptions, captions, preferred font, etc.)?*

10.  Do you know of any training resources or materials that currently help youth engage as research partners?

*a.* *Probe: What are these resources?*

*b.* *Probe: What do you like about these resources? Are there anything you wish these resources included?*

c. *Probe: How can we make our training materials unique?*

11.  What impact do you think these training materials could have on engagement of youth with neurodevelopmental disabilities in research that are relevant to them?

*a.* *Probe: What do you think would be the benefits of engaging more youth as research partners?*

12.  [Summarize key points from the session] Is there anything that we may have missed that you would like to add?

13.  What is your takeaway message from today’s session? What is one thing to prioritize in our training materials?

14.  Do you have any questions for me?
