# Supplementary material for: Youth engagement in research: exploring training needs of youth with neurodevelopmental disabilities
Source: Res Involv Engagem. 2023 Jul 10;9:50. doi: 10.1186/s40900-023-00452-3 (PMC10332095; doi:10.1186/s40900-023-00452-3)
Supplement: Supplementary file 2 — Additional file 2. Virtual symposium summary. [file 40900_2023_452_MOESM2_ESM.pdf]

Youth Engagement in Research

VIRTUAL SYMPOSIUM SUMMARY

OBJECTIVES

DAY 1:
September 15, 2021
Identify training needs and materials

DAY 2:
September 25, 2021
Priority setting on training needs and materials

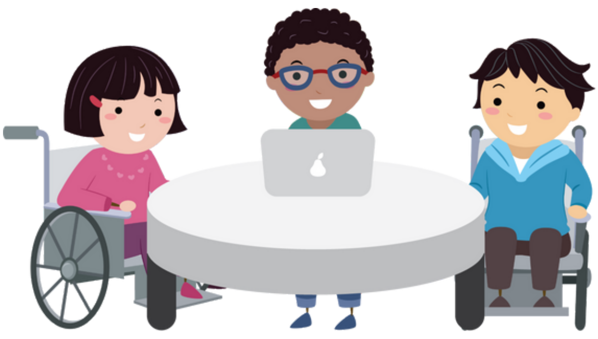

DEMOGRAPHIC OF ATTENDEES

Total Number = 17

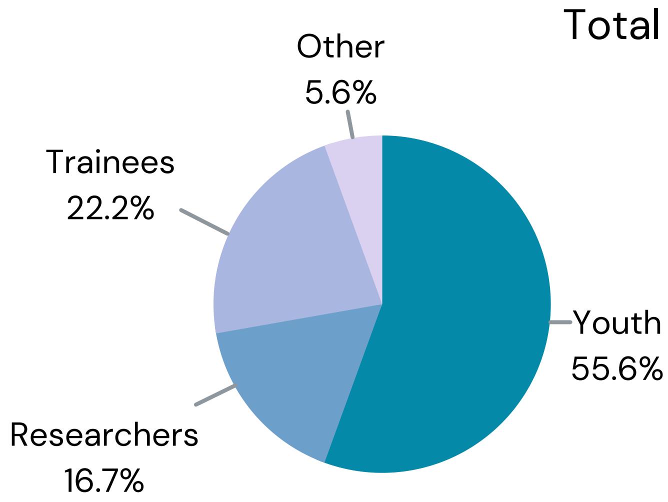

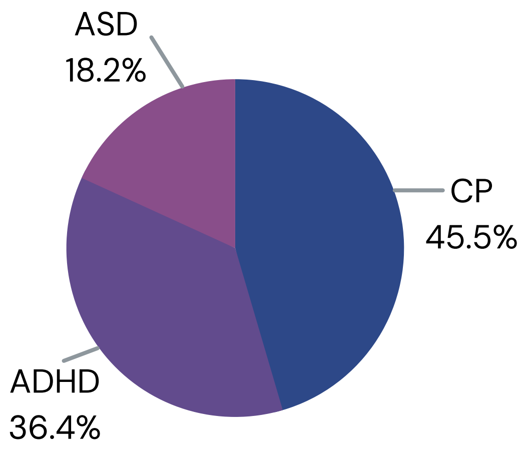

*Note some individual have multiple roles/conditions

SUMMARY OF DAY 1

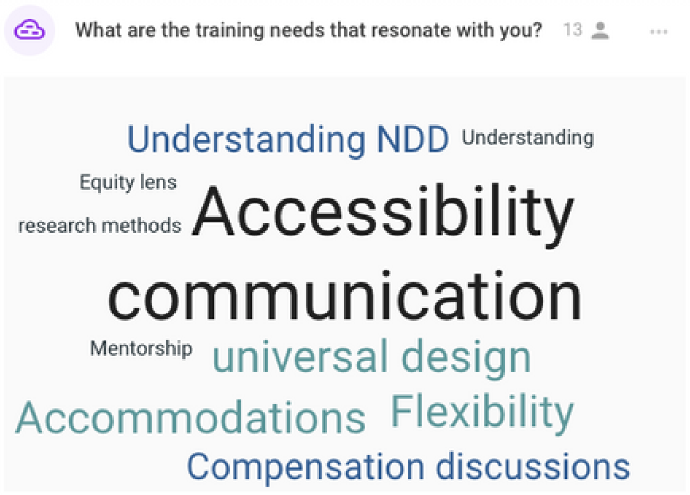

Potential Formats:

- Online and interactive modules
- Include quizzes, activities, and reflections
- Simulations (e.g., scenarios + solutions)
- Videos with a person speaking 'to you'
- Whiteboard animations with narrator voiceover
- Mentorship (personal check-ins)

SUMMARY OF DAY 2

PRIORITIZED TRAINING TOPICS

1. **Communication training between youth and researchers**
2. **Research roles and responsibility**
3. **Finding research partnership opportunities***
4. Fostering authentic and impactful engagement*
5. Creating a safe space for research
6. Understanding the research process

*Tied initially and re-voted.

See the summary of the content and delivery method discussions for the top 3 training topics below.

THANK YOU!

Your valuable input will help us develop co-training opportunities for researchers and youth with neurodevelopmental disabilities (ages 18-25) to help foster equal and active research partnerships.

Summary: Day 2 Virtual Symposium Discussions

Topic	Content/Curriculum	Delivery Method
**Communication training between youth and researchers**	• Communication methods<br>○ Establish at upfront method in the beginning of partnership<br>○ Be consistent among the team<br>○ Combination of methods for different purpose (e.g., email for whole group, text for clarification)<br>○ Other platforms (more accessible):<br>○ Text, Messenger, etc.<br>○ 1-on-1 conversation for accessibility needs (keep track on a spreadsheet)<br>○ Use doodle polls and other polls/tools that are easy to fill out<br>• How to navigate effective communication<br>○ Co-designed terms of reference between youth and researchers that outline the ethics, expectations, confidentiality<br>○ Clear list outlining who to directly contact for specific inquiries (e.g., compensation, accessibility, etc.)<br>○ Cut down the use of acronyms and use plain language	• Interactive modules, chat rooms, discussion boards for questions (asynchronous)<br>• Synchronous sessions<br>• Checklist for youth and researcher<br>• Choose your own adventure simulation of communication scenarios<br>• Infographic (used on multiple platforms)

<table>
<tr><td>Research roles and responsibilities</td><td>Intake survey/conversation at the beginning of the research project
Involvement matrix – define roles and extent of responsibilities through research cycle
Breakdown roles throughout the research process: Recruitment, execution of study, knowledge translation
Present alternative way of contributing to the project (e.g., directing researchers to certain information and resources) Openness to different needs and ways to communicate
Awareness that roles can change and adapt over time.
Not shy away from discovering new roles and skills
Encourage leadership</td><td>CHILD-BRIGHT has interactive electronic infographic for patient partner engagement
OBI Framework – can be simplified
Discussion forums
Synchronous or live meeting opportunities
Self-directed at your own pace online modules
Bottom-up approach
Ensuring resources (documents, websites, videos) are accessible and can be adaptable to multiple formats/needs
Mentorship opportunity for people to connect, share experience of “what is working well”
Skill-building workshops for different responsibilities and research partner may have</td></tr>
<tr><td>Finding research partnership opportunities</td><td>Effort to promote research partnership opportunities in BC: www.reachbc.ca
Clarity in postings
Currently very insular
Make more public and centralized</td><td>Student thesis project
Difficult in terms of feasibility – interagency and collaboration needs to happen
Smaller collaboration in the beginning, then get larger</td></tr>
</table>

	○ Lack of visibility of projects (have to go to university departments to find projects)<br>○ Communicate clearly what exactly researchers are looking for<br>● Lack of diversity → need to change the way of recruitment<br>○ Researcher make space more comfortable<br>● Guideline for researchers<br>○ Being careful about postings that could be triggering<br>● Provide more virtual engagement opportunities (national as opposed to local/provincial)<br>● Two way profiles (youth and researchers) – research interest, willingness to travel. Similar to a “dating” app but for research.<br>● How to look? Where to look?<br>○ Terms to look for when searching (e.g., researcher, research provider, research consultant)	● Co-learning – what youth would want to see in a write-up<br>● Mentorship to help find opportunities, attending initial meetings with you (support person)<br>● More experienced youth may have too many opportunities/knowledgeable about opportunities → place to share with other youth interested
