# Supplementary material for: Youth engagement in research: exploring training needs of youth with neurodevelopmental disabilities
Source: Res Involv Engagem. 2023 Jul 10;9:50. doi: 10.1186/s40900-023-00452-3 (PMC10332095; doi:10.1186/s40900-023-00452-3)
Supplement: Supplementary file 3 — Additional file 3. Partner responses to the public and patient engagement evaluation tool (PPEET). [file 40900_2023_452_MOESM3_ESM.docx]

Table 5. Partner responses to the PPEET

|  |  | n | % |
| --- | --- | --- | --- |
| 1. What perspective do you bring to the execution phase for the Youth Engagement in Research (YER) Study? | | | |
|  | Patient/patient advisor/patient partner | 4 | 80% |
|  | Family member/caregiver | 1 | 20% |
| 2. How long have you been working with the YER Study as a research partner? | | | |
|  | 6 - 12 months | 3 | 60% |
|  | 1 - 2 years | 2 | 40% |
| 3. I have a clear understanding of the purpose of the execution phase for the YER Study. | | | |
|  | Strongly agree | 3 | 60% |
|  | Agree | 2 | 40% |
| 4. The supports I need to participate in the execution phase of the YER Study are available (e.g., flexibility, accessibility). | | | |
|  | Strongly agree | 4 | 80% |
|  | Agree | 1 | 20% |
| 5. I have enough information to be able to carry out my role. | | | |
|  | Strongly agree | 3 | 60% |
|  | Agree | 2 | 40% |
| 6. I am able to express my views freely. | | | |
|  | Strongly agree | 3 | 60% |
|  | Agree | 2 | 40% |
| 7. I feel that my views are heard. | | | |
|  | Strongly agree | 4 | 80% |
|  | Agree | 1 | 20% |
| 8. A wide range of views on discussion topics is shared. | | | |
|  | Strongly agree | 3 | 60% |
|  | Agree | 1 | 20% |
|  | Neither agree or disagree | 1 | 20% |
| 9. This execution phase of the YER Study is achieving its stated objectives. | | | |
|  | Strongly agree | 2 | 40% |
|  | Agree | 2 | 40% |
|  | Neither agree or disagree | 1 | 20% |
| 10. I am confident that the research team takes the feedback provided in the execution phase of the YER Study into consideration. | | | |
|  | Strongly agree | 4 | 80% |
|  | Agree | 1 | 20% |
| 11. I think that the work in the execution phase of the YER Study makes a difference to the work of the research team. | | | |
|  | Strongly agree | 4 | 80% |
|  | Neither agree or disagree | 1 | 20% |
| 12. As a result of my participation in the execution phase of the YER Study, I am better informed about youth engagement in research. | | | |
|  | Strongly agree | 4 | 80% |
|  | Agree | 1 | 20% |
| 13. Overall, I am satisfied with the execution phase of the YER Study. | | | |
|  | Strongly agree | 3 | 60% |
|  | Agree | 2 | 40% |
| 14. The execution phase of the YER Study is a good use of my time. | | | |
|  | | | |
|  | Strongly agree | 4 | 80% |
|  | Agree | 1 | 20% |
